# Evaluation of the Role of Claudin-4 Antigen Overexpression in Triple-Negative Breast Cancer Patients: A 5-Year Survival Analysis 

**DOI:** 10.30699/ijp.2025.2040168.3350

**Published:** 2025-03-10

**Authors:** Maryam Soltan, Azar Naimi, Razieh Hafez Forghan, Marjan Mansourian

**Affiliations:** 1 *Department of Pathology, Reproductive Sciences and Sexual Health Research Center, Isfahan University of Medical Sciences, Isfahan, Iran*; 2 *School of Medicine, Isfahan University of Medical Sciences, Isfahan, Iran*; 3 *School of Health, Department of Biostatistics and Epidemiology, Isfahan University of Medical Sciences, Isfahan, Iran*

**Keywords:** Breast Neoplasms, Claudin-4, Biomarkers, Prognosis, Neoplasm Metastasis

## Abstract

**Background & Objective:**

Breast cancer (BC) can be categorized into 4 groups based on molecular and pathological evidence: Luminal A, Luminal B, HER2+ tumors, and triple-negative breast cancer (TNBC). TNBC has a poorer survival rate and a higher chance of recurrence and metastasis compared to other BC types, primarily due to its challenging treatment course. Claudin 4 (CLDN4), a transmembrane protein in tight junctions between cells, has been linked to poor prognosis and faster disease progression in these malignancies.

**Methods:**

Patients previously diagnosed with TNBC and tested for CLDN4 overexpression were contacted for follow-up and to determine disease outcomes. The current health status, cause, and time of death (if applicable) were recorded. Patient files were accessed to obtain information on age, tumor size and grading, lymph node involvement, metastasis, Ki67, and CLDN4 expression.

**Results:**

Patients with high CLDN expression showed a significantly lower mortality rate. However, after controlling for other covariates, the hazard ratio (HR) was 0.48 (95%CI= [0.13 – 1.27]) in the crude model for survival, 0.54 (95%CI = [0.2 – 1.43]) when adjusted for age at diagnosis, and 0.58 (95%CI = [0.18-1.82]) when adjusted for other covariates. CLDN4 was also not correlated with tumor metastasis (HR=0.64, p=0.203, in the crude model; HR=0.52, p=0.409, when adjusted for other covariates). Patients in the CLDN4 high group had a significantly higher number of tumors >2cm.

**Conclusion:**

Although previous studies have shown that CLDN4 overexpression worsens TNBC prognosis and increases metastasis or recurrence, the current study found no such association.

## Introduction

Breast cancer (BC) reigns as the most common malignancy affecting women globally, posing a significant public health burden (1, 2). Each year, over 1.3 million new cases are diagnosed, tragically resulting in roughly 460,000 deaths (3). The relentless pursuit of earlier BC detection and treatment strategies remains a paramount focus. Regardless of BC subtype, early identification and appropriate intervention are crucial for improved prognosis and reduced mortality rates (4). Mammography is the current gold standard for screening and biopsy, with definitive diagnosis relying on pathological analysis (4). This analysis can additionally identify various biomarkers expressed within malignant tissues. Biomarkers hold immense value in predicting disease course and prognosis (5). BC classification hinges on a subtyping approach based on the expression levels of critical receptors: estrogen receptor (ER), progesterone receptor (PR), Ki-67, and HER2 (5). As such, BC is grouped into Luminal A (ER-positive (ER+), PR positive, HER2 negative, Ki67 <14%) (6), Luminal B‐like (HER2‐negative) tumors (ER+, HER2 negative, and Ki67 ≥14%), Luminal B‐like (HER2‐positive [HER2+]) tumors (ER+, HER2+, any Ki67 level), HER2+ (non‐luminal) tumors (HER2+ and ER and PR negative) and finally triple‐negative (ductal) tumors are defined as ER, PR, and HER2 negative (7, 8).

Treatment strategies for BC are heavily influenced by its underlying genetic makeup and associated biomarkers. Broadly, treatment regimens encompass systemic chemotherapy alongside targeted therapies like recombinant monoclonal antibodies and PARP inhibitors (9). Compared to other BC subtypes, TNBC exhibits a poorer survival rate, a higher risk of recurrence, and a more challenging treatment course due to its limited targetability (10, 11).

Claudins, transmembrane proteins forming tight junctions between cells, play a vital role in regulating intercellular transport and maintaining epithelial cell polarity (12, 13). Claudin 4 (CLDN4) is one of the significant CLDNs involved in tight junctions and is associated with many different malignancies, as it can influence the metastasis rate of malignancies. Its overexpression has been reported in gastric (14), pancreatic (15), colorectal (16), and BC (especially TNBC) (17, 18). Studies suggest that CLDN4 overexpression correlates with poor prognosis and accelerated disease progression in these cancers. However, the precise impact of CLDN4 on long-term mortality in BC remains a subject of ongoing investigation and conflicting reports. Building upon a previous study that assessed CLDN4 overexpression in Isfahan's TNBC population, this current investigation delves into the long-term outcomes and mortality rates associated with CLDN4 expression.

## Materials and Methods

This retrospective study recruited participants previously diagnosed with TNBC in Isfahan, Iran. Patients were categorized into two groups based on CLDN4 expression levels, which were determined in a prior investigation (Figure 1). Inclusion criteria encompassed prior participation in the initial study and obtaining informed consent. Incomplete patient records served as the sole exclusion criterion.

The study protocol was thoroughly explained to patients or their designated next of kin after telephone contact. Informed consent for participation was then obtained from the participants themselves if they were alive, or from their immediate family if they had passed away. Current health status was assessed, including deceased patients' cause and time of death. Demographic and clinical data, including age at diagnosis, tumor size and grade, lymph node involvement, metastasis, Ki-67 index, and CLDN4 expression, were retrieved from medical records.

In statistical analysis categorical data are presented as frequencies with percentages (%), while scale data were expressed as means ± standard deviation (SD). Statistical analysis was performed using SPSS® for Windows® (version 20) software. Chi-square or Mann-Whitney U tests were employed to analyze qualitative data, while independent T-tests evaluated quantitative data. Five-year overall survival was estimated using comparative survival curves and Kaplan-Meier survival analysis with a log-rank test implemented in STATA® (version 14). A P-value of less than 0.05 was considered statistically significant.

## Results

As shown in table-2, patients in the CLDN high group and CLDN low group had no significantly different in case of mortality, recurrence, and metastasis(P>0.05).

As depicted in Table 3, the log-rank test was utilized to evaluate the outcomes. Model 1 was a crude model, Model 2 was adjusted for the age at the time of diagnosis, and Model 3 was adjusted for other covariates. No significant difference was observed in the 5-year overall survival, metastasis, and recurrence rates for breast cancer between the two groups with high and low CLDN expression. This observation remained consistent even after adjusting for age at diagnosis, type of treatment, and other covariates (Table 3).

The hazard ratio (HR) for 5-year overall survival was 0.48 (95% CI = [0.13 – 1.27]) in the crude model. When adjusted for age at the time of diagnosis, it changed to 0.54 (95% CI = [0.20 – 1.43]), and to 0.58 (95% CI = [0.18-1.82]) when adjusted for other covariates. In the model for metastasis, the HR was 0.64 (95% CI = [0.23-1.27]) in the crude model, 0.6 (95% CI = [0.21-1.67]) when adjusted for age, and 0.52 (95% CI = [0.15 – 1.52]) when adjusted for other covariates. The HR for recurrence rates was 0.86 (95% CI = [0.83-8.96]) in the crude model, 0.84 (95% CI = [0.78-9.32]) when adjusted for age, and 0.83 (95% CI = [0.71-1.02]) when adjusted for other covariates.

It should be noted that although CLDN4 overexpression, based on the HR, exhibited a protective effect on survival, recurrence, and metastasis, it did not reach statistical significance due to the small sample size and consideration of other covariates. A larger sample size makes statistical significance more likely to be achieved.

**Table 1:** Demographic and clinical variables. Chi-square was used for categorical and independent T-tests, and the Mann-Whitney test was used for scale variables. Statistical significance is defined as having a p-value less than 0.05.

Characteristic	CLDN high (n = 58)	CLDN Low (n = 14)	P-value
Age at diagnosis, mean year (SD)	54.3(11.6)	58.6(12.7)	0.223
Tumor grade, N (%)			
Grade 1	2 (3.4)	2 (14.3)	0.281
Grade 2	15 (25.9)	3 (21.4)	
Grade 3	41 (70.7)	9 (64.3)	
Disease Stage, N (%)			0.154
1	16 (24.6)	6 (42.9)	
2	25 (43.1)	4 (28.6)	
3	13 (22.4)	1 (7.1)	
4	4 (6.9)	3 (21.4)	
Type of surgery received, N (%)			
Lumpectomy	39 (67.2)	10 (71.4)	0.517
Mastectomy	19 (32.8)	4 (28.6)	
No surgery	0 ()	0 ()	
lymph node involvement, N (%)	18 (31.0)	4 (28.6)	0.567
Tumor size (cm), N (%)			
≤ 2	16 (27.6)	6 (42.9)	0.368
>2	42 (72.4)	8 (57.0)	
Cancer history in the family, N (%)			
No	30 (51.7)	6 (42.9)	0.587
Breast Cancer	15 (25.9)	3 (21.4)	
Other cancers	13 (22.4)	5 (35.7)	
Treatment type			0.560
Chemotherapy and radiotherapy	51 (87.9)	12 (85.7)	
Chemotherapy	7 (12.1)	2 (14.3)	
Ki-67 expression, mean % (±SD)	46.55(±20.2)	42.79(±20.2)	0.617
Disease history, N (%)			
No history	37 (63.8)	11 (78.0)	0.234
CVD, hypertension, or diabetes	21 (34.5)	3 (21.4)	

**Table 2:** Outcomes. Chi-square was used Statistical significance is defined as having a p-value less than 0.05.

Characteristic	CLDN high (n = 58)	CLDN Low (n = 14)	p-value
Death, N (%)	14 (24.1)	6 (42.8)	0.143
Recurrence, N (%)	4 (6.9)	1 (7.1)	0.673
Metastasis, N (%)	15 (25.9)	5 (35.7)	0.333

**Table 3:** Multivariable analysis of breast cancer-specific 5-year overall survival, breast cancer metastasis, and breast cancer-specific recurrence by Claudin expression status. Model 1 is crude; Model 2 is adjusted by age at the time of diagnosis and treatment course; Model 3 is adjusted by age and other covariates, as described in Table 1. The hazard ratio is calculated using the log-rank test, and statistical significance is defined as *p* < 0.05.

		Model 1	Model 2	Model 3
Breast cancer-specific 5-year overall survival	HR (%95 CI)	0.48 (0.13, 1.27)	0.54 (0.20, 1.43)	0.58 (0.18, 1.82)
	P-value	0.123	0.806	0.338
Breast cancer metastasis	HR (%95 CI)	0.64 (0.23, 1.27)	0.60 (0.21, 1.67)	0.52 (0.15, 1.52)
	P-value	0.203	0.331	0.409
Breast cancer-specific recurrence	HR (%95 CI)	0.86 (0.83, 8.96)	0.84 (0.78, 9.32)	0.83 (0.70, 10.2)
	P-value	0.967	0.856	0.675

## Discussion

 This study focused on the CLDN4 gene expression levels in previous BC patients. Patients were categorized into two groups based on their CLDN4 expression. The findings revealed that the mortality rate was significantly higher in the CLDN4 low-expression group. However, neither group exhibits significant differences in demographic characteristics such as age, disease stage, tumor grade, treatment regimens employed, and Ki67 levels. Survival analysis demonstrated that although the mortality rate was higher in the CLDN4 low expression group, there was no significant difference in the 5-year overall survival between the two groups, even after adjusting for covariates. Furthermore, survival analysis and hazard ratio for recurrence and metastasis also showed no significant difference between the two groups. Our study indicates that CLDN4 expression has no significant impact on patients' 5-year overall survival, metastasis, or recurrence of BC.

CLDN4, a tight junction protein belonging to the CLDN family, has emerged as a potential biomarker for malignant tumors. Prior in vivo studies have linked CLDN4 overexpression to accelerated disease progression, advanced staging, and poor prognosis in BC cells. Ma et al. demonstrated that elevated CLDN4 expression in MCF-7 BC cell lines enhanced proliferation and migration while reducing apoptosis (18). Conversely, Kolokytha et al. reported that CLDN4 overexpression correlated with shorter disease-free survival and overall survival at five years, specifically in TNBC patients, but only increased Ki-67 proliferation index and tumor stage in luminal BC patients (19). A critical distinction between our study and Kolokytha et al.'s work lies in their focus on ductal carcinoma, whereas we recruited TNBC patients irrespective of tumor location. 

Abd-Elazeem et al. investigated CLDN4 overexpression in TNBC patients with primary invasive ductal carcinoma, identifying a positive and significant correlation with age, tumor size, grade, lymph node involvement, metastasis, and Ki-67 expression (20). Their findings further demonstrated that higher Ki-67, a marker of poor prognosis, co-occurred with CLDN4 overexpression, suggesting CLDN4 as a valuable prognostic indicator in TNBC. Szasz et al. bolstered the role of CLDN4 overexpression in TNBC prognosis by highlighting its potent predictive power alongside CLDN3, CLDN7, and E-cadherin in survival analyses. They reported that CLDN4 and E-cadherin accurately predicted relapse-free survival in a validation cohort at five years, with efficacy across luminal and ductal carcinomas (21). Additional studies have corroborated the association between CLDN4 overexpression and worse prognosis in TNBC patients (22-25). 

However, our findings diverge from previous research. We observed no correlation between CLDN4 overexpression and Ki-67, staging/grading, metastasis, recurrence, or five-year overall survival rates. This discrepancy might be partially attributed to our limited sample size (14 patients in the low CLDN4 group), variable time intervals since diagnosis, and incomplete access to clinical data. Socioeconomic factors also played a role. Many patients belonged to lower socioeconomic classes, resulting in infrequent follow-ups and incomplete treatment regimens for TNBC and other co-morbidities, leading to unrelated mortality.

Furthermore, genetic differences within the Iranian population may be another contributing factor. Cancer susceptibility and prognosis are heavily influenced by patient genetics. Prior studies have shown that ethnicity can impact five-year overall survival rates (26-28). Unfortunately, research on gene expression in the Iranian population remains limited, hindering robust conclusions. While the influence of ethnicity on biomarker-prognosis associations remains unclear, we advocate for further studies exploring this topic within the Iranian population.

## Conclusion

In conclusion, this retrospective study examining the impact of CLDN4 expression on long-term outcomes in triple-negative breast cancer patients from Isfahan, Iran, did not find a significant association between CLDN4 levels and five-year overall survival, metastasis, or recurrence rates. Although the mortality rate was higher in the CLDN4 low expression group, the differences were not statistically significant after adjusting for potential confounders. These findings diverge from prior research linking CLDN4 overexpression to poorer prognosis, potentially due to limited sample size, incomplete data, and genetic variations within the Iranian population. Further larger-scale studies with standardized follow-up protocols and consideration of ethnic differences are warranted to clarify the prognostic value of CLDN4 in diverse populations and its potential utility as a biomarker for personalized treatment strategies.

## Data Availability

Data are available upon reasonable request from the corresponding author.
